# Study and Treatment of Oral Squamous Cell Carcinoma—Insights and Perspectives

**DOI:** 10.3390/cancers15204968

**Published:** 2023-10-13

**Authors:** Samer George Hakim, Yu-Xiong (Richard) Su

**Affiliations:** 1 Department of Oral and Maxillofacial Surgery, Head and Neck Cancer Center, University Hospital Schleswig-Holstein (Campus Lübeck), D-23562 Luebeck, Germany; 2 Department of Oral and Maxillofacial Surgery, Helios Medical Center, D-19055 Schwerin, Germany; 3 Division of Oral and Maxillofacial Surgery, Faculty of Dentistry, The University of Hong Kong, Hong Kong; richsu@hku.hk

The prevalence of oral squamous cell carcinoma (OSCC) has increased in recent decades, and its impact on the health system has become a new aspect. In addition to the well-known exogenous noxae like nicotine and alcohol consumption, as well as some distinct nutritional habits such as betel nut chewing and virus infection by the human papilloma virus (HPV), its transfer via sexual practices augmented the recent increase in the incidence of oropharyngeal cancer [1]. Finally, the development and establishment of immune-suppressive treatments for autoimmune diseases and in patients with organ transplants enhanced the potential development of OSCC in precursor lesions. Currently, we are dealing with more than 377,000 new cases and 177,000 deaths related to lip and oral cavity tumors worldwide in 2020, according to the Global Cancer Observatory report.

In parallel, our understanding of the initiation and development of oral cancer has also changed substantially. The molecular basis for the epithelial invasion mechanisms, including the epithelial–mesenchymal transition (EMT) and remodeling of the extracellular matrix (ECM), not only intensified our knowledge concerning the underlying pathology but also brought more sophisticated diagnostic methods and prognostic tools, as well as promising therapeutic options for patients with OSSC [2,3,4].

The original research and review articles included in this Special Issue elucidate the diversity of molecular mechanisms involved in the onset, invasion, and progression of OSCC, with particular attention paid to the role of HPV infection and multimodal treatment. Finally, a state-of-the-art review of modern 3D-based microsurgical reconstruction following ablative oncologic surgery of the head and neck region enriches the scope of this Special Issue [5,6]. These articles highlight special aspects in the diagnosis and treatment of OSCC and provide an orientation in the management of patients with OSCC in terms of diagnosis, patient care, and quality of life.
